# CD74-Targeting Antibody–Drug Conjugate Enhances Immunosuppression of Glucocorticoid in Systemic Lupus Erythematosus

**DOI:** 10.3390/ijms262311761

**Published:** 2025-12-04

**Authors:** Qizhen Du, Shengtao Yao, Yuying Huang, Jia Zhang, Wangmo Sonam, Xiao Lu, Jichao Yang, Shipeng Cheng, Ran Wang, Jiefang Xu, Liyan Ma, Yu Liu, Guanghao Wu, Jing Zhang, Xuelei Wang, Wei Lu, Zhiyang Ling, Chunyan Yi, Bing Sun

**Affiliations:** 1 Key Laboratory of Multicellular Systems, CAS Center for Excellence in Molecular Cell Science, Shanghai Institute of Biochemistry and Cell Biology, University of Chinese Academy of Sciences, Chinese Academy of Sciences, 320 Yueyang Road, Shanghai 200031, China; 2 Shanghai Pharmaceuticals Holding Co., Ltd., Halei Road 898, Shanghai 201203, China; 3 School of Life Science and Technology, ShanghaiTech University, Shanghai 201210, China; 4 Division of Life Sciences and Medicine, University of Science and Technology of China, Hefei 230052, China; 5 Shanghai Institute of Nutrition and Health, Chinese Academy of Sciences, University of Chinese Academy of Sciences, 320 Yueyang Road, Shanghai 200031, China

**Keywords:** antibody-drug conjugates (ADC), glucocorticoid (GC), anti-CD74 antibody, systemic lupus erythematosus (SLE), budesonide

## Abstract

Glucocorticoid drugs (GCs), while effective in systemic lupus erythematosus (SLE), cause severe systemic side effects due to lack of tissue-specificity. To overcome this bottleneck, we developed a CD74-directed antibody–drug conjugate (Bud-ADC) to deliver budesonide, a potent GC drug, selectively to target CD74-expressing immune cells (e.g., B cells, dendritic cells), which play an important role in SLE pathogenesis. Bud-ADC combines a cross-species anti-CD74 antibody with budesonide via a cleavable linker, enabling immunosuppression on targeted cells. In vitro, Bud-ADC selectively inhibited CD74-high immune cell activation and cytokine production. In two SLE mouse models, Bud-ADC significantly alleviated disease hallmarks—reducing autoantibodies, splenomegaly, and kidney damage—while showing superior efficacy to free budesonide at equivalent doses. The therapeutic effects involved both direct targeting of CD74-high immune cells and indirect modulation of T cell responses despite low CD74 expression. This study establishes CD74-targeted ADC as a novel strategy to enhance GC efficacy in SLE, aiming at minimizing off-target toxicity while maintaining broad immunosuppressive activity. The translatable design supports further preclinical and clinical development for autoimmune diseases.

## 1. Introduction

Antibody–drug conjugates (ADCs) are a class of macromolecular biologics typically composed of a monoclonal antibody targeting specific antigens on cell surface, a cytotoxic small-molecule payload, and a covalent chemical linker connecting them together [1]. ADCs represent a prominent direction in tumor therapeutics. The primary mechanism of action involves monoclonal antibodies targeting proteins highly expressed on tumor cells but absent or minimally expressed in normal tissues. Through endocytosis, cytotoxic payloads are specifically delivered to tumor cells, achieving tumor cell killing while sparing normal tissues [2,3]. By combining the high specificity of monoclonal antibodies with the potent cytotoxicity of small molecules, ADCs enable precise and efficient antitumor effects. As of 2025, 15 ADCs have been approved for treating various hematologic malignancies and solid tumors. Over 100 ADC candidates are currently in clinical development, among which the majority are aimed at cancer treatment [1,4,5].

While ADC development remains predominantly focused on oncology, researchers have begun exploring their applications in non-cancer diseases. As early as 2002, Everts et al. proposed the concept of using E-selectin to deliver dexamethasone to activated endothelial cells [6]. Subsequent studies have investigated ADCs’ potential in chronic inflammatory diseases (e.g., atherosclerosis, ulcerative colitis), autoimmune disorders (e.g., rheumatoid arthritis, myasthenia gravis, systemic sclerosis), bacterial infections, and renal/hepatic diseases [7,8,9,10,11,12,13,14]. The design of these non-oncological ADCs relies on understanding disease pathogenesis, selecting appropriate cellular targets, and developing suitable payloads. Unlike conventional tumor-targeting ADCs designed for cell killing, non-cancer ADCs typically modulate specific cellular processes to influence pathogenic cell functions without inducing cytotoxicity. Most non-oncological ADCs remain in early preclinical exploration stages, requiring further investigation [14,15].

Systemic lupus erythematosus (SLE) is a chronic autoimmune disease with high morbidity and mortality, predominantly affecting women [16]. Its pathogenesis involves complex immune dysregulation affecting multiple organ systems [17]. At the cellular level, SLE is driven by interactions between adaptive and innate immunity, involving T cells, B cells, monocytes, macrophages, dendritic cells (DCs), plasmacytoid DCs (pDCs), neutrophils, etc. Those multiple cell types cause autoantibody production, cytokine upregulation, complement activation, immune complex deposition, and ultimately inflammation and tissue damage [18,19,20]. Glucocorticoids (GCs) have been mainstays in SLE treatment for decades [21]. While GCs improve patients’ quality of life, their systemic side effects—including Cushingoid changes, osteoporosis, insulin resistance, hyperglycemia, cardiovascular complications, and cataracts—severely limit clinical utility [22]. These limitations arise from the ubiquitous expression of glucocorticoid receptors regulating diverse biological processes [23].

To overcome GC-related toxicity, novel targeted delivery systems are being developed to enhance drug specificity, bioavailability, and pharmacokinetics [24]. ADCs, as advanced drug delivery platforms, offer an ideal solution for GCs when paired with appropriate targets. Recent studies have explored GCs (e.g., dexamethasone) as ADC payloads for delivery to endothelial cells and macrophages [13,25]. In recent years, pharmaceutical companies such as AbbVie, Regeneron, and Merck have successively developed several ADCs for the targeted delivery of glucocorticoid drugs. The targets involved include TNFα, CD74, CD70, PRLR, etc., and the payloads include derivatives of potent glucocorticoid drugs such as budesonide and fluticasone propionate or other new glucocorticoid drugs [8,9,10,12,26,27]. Judging from the current development status of GC-ADCs [28,29], this type of drug has shown good therapeutic potential in chronic inflammatory diseases, but the drugs entering clinical development have not been smoothly promoted, indicating that there is still need of promotion, including better target selection, drug design, indication selection, and more detailed preclinical research.

Given the central role of multiple immune cell types in SLE pathogenesis, broad suppression of their functions may achieve optimal therapeutic effects [18]. To enable GCs to act widely across immune cell populations while avoiding off-target effects, we propose targeting membrane proteins with high expression and endocytic capacity on immune cells. As observed from analysis of public datasets and the literature, CD74 has been preliminarily identified as a promising target for GC-ADC development. CD74 is a type II transmembrane glycoprotein, initially recognized for its critical chaperone role in MHC-II antigen presentation [30]. Subsequent studies have revealed its diverse immunomodulatory functions [31,32,33,34]. CD74 is highly expressed in multiple immune cell types, including B cells, DCs, pDCs, and macrophages [35]—all of which mediate distinct aspects of systemic lupus erythematosus (SLE) pathogenesis. Additionally, CD74 is highly suitable for ADC development due to its robust endocytic capacity [36,37,38]. Many therapies targeting certain immune cell types or related pathways have entered clinical development, with biologics targeting B cells or the type I IFN pathway (primarily derived from pDCs) already approved for SLE treatment [39,40,41,42]. Therefore, leveraging CD74 as a therapeutic target to deliver potent GC drugs to simultaneously suppress these key pathogenic immune cell types in SLE, compared to single-pathway modulation, theoretically could exert broader therapeutic effects, and may avoid the side effects of traditional GC drugs as well.

Herein, we validated the feasibility of developing a glucocorticoid–antibody conjugate (GC-ADC) targeting CD74. Using our in-house rabbit monoclonal antibody platform [43], we generated a cross-reactive anti-CD74 antibody that binds to both human and mouse CD74. This antibody was then conjugated with budesonide, a potent GC drug, to form a GC-ADC designated as Bud-ADC, whose therapeutic potential and safety were subsequently evaluated. As expected, the Bud-ADC showed excellent immunosuppressive activity both in vitro and in vivo, with particularly superior efficacy compared to free budesonide in murine SLE models.

## 2. Results

### 2.1. CD74 Is Highly Expressed on Certain Immune Cell Types and Capable of Rapid Internalization

**Figure 1:**
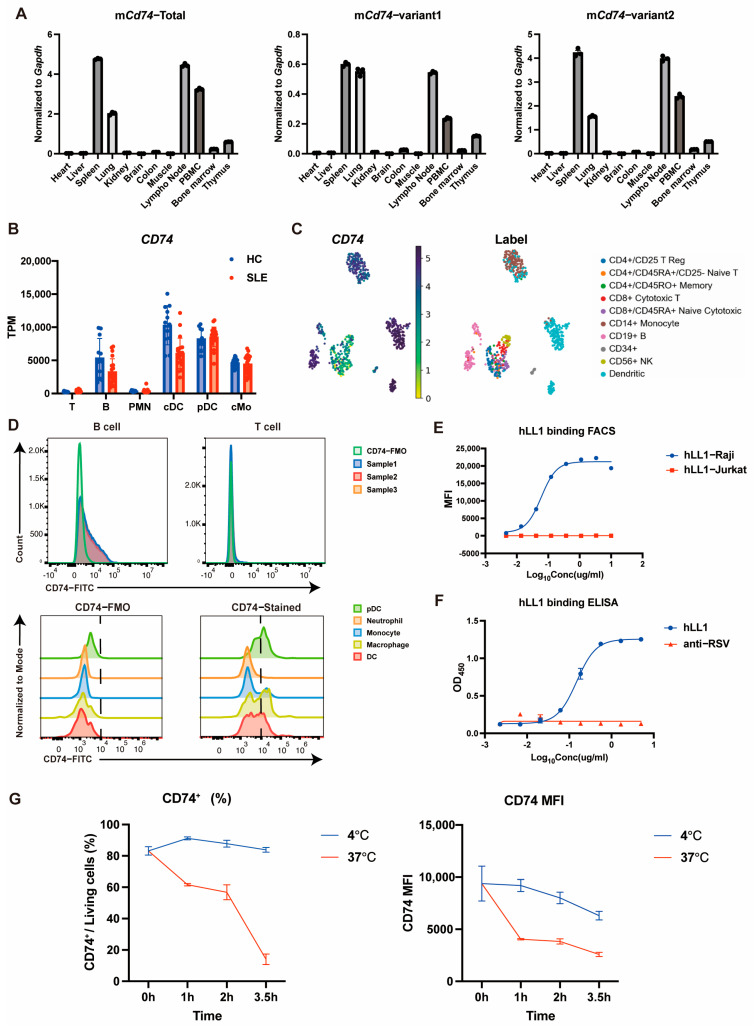
CD74 expression pattern and internalization property analysis. (**A**) Mouse *Cd74* RNA expression in different organs or tissues detected by quantitative RT-PCR; (**B**) Expression profiling of *CD74* in different immune cell types from Healthy Control (HC) and SLE patients, GSE149050; (**C**) Single cell expression profiling of *CD74* in different immune cell types of human PBMCs, 10xGenomics Fresh68K PBMCs; (**D**) CD74 expression pattern of mouse splenocytes detected by flow cytometry; (**E**,**F**) Binding property of hLL1 measured by flow cytometry (**E**) and ELISA (**F**); (**G**) Internalization assay of hLL1 measured by flow cytometry. The results in (**A**,**F**,**G**) are presented as the means ± SEM. Data are representative of two or three independent experiments.

To ascertain the expression profiles of CD74 on immune cell populations, we systematically examined the expression pattern of CD74 across different organs and various cell types. Initially, the expression of *Cd74* in murine major organs was determined by quantitative RT-PCR, including its two variants. It was interesting to observe that total *Cd74* and its two variants were highly expressed in immune cell-abundant organs such as the spleen, lymph nodes, and lungs. Notably, *Cd74* was also highly expressed in PBMCs (Peripheral blood mononuclear cells) (Figure 1A). These results demonstrate that CD74 is an ideal target expressed by immune cells. Given the importance of immune cell regulation in the pathogenesis of SLE, we further investigated the specific immune cell types exhibiting the highest expression of *CD74* by analyzing publicly available RNA sequencing datasets, including those from immune cells of SLE patients and healthy individuals (GSE149050, 10xGenomics Fresh68K PBMCs). Consistent with previous findings, cDCs, pDCs, B cells and monocytes were found to express high-level *CD74* (Figure 1B,C). To further confirm this observation, we assessed the protein level of CD74 in different immune cell types isolated from mouse spleen by flow cytometry. The results revealed that B cells, DCs, pDCs, and macrophages expressed higher levels of CD74, while T cells and neutrophils showed minimal expression (Figure 1D).

To test the internalization properties of CD74, we employed a well-characterized anti-human CD74 antibody hLL1 [44]. We evaluated its binding activity by flow cytometry and ELISA, which demonstrated that hLL1 showed high affinity against high-CD74-expressing Raji cells and immobilized human CD74 protein (Figure 1E,F). The internalization kinetics was measured by incubating hLL1-pre-bound Raji cells at 4 °C and 37 °C, respectively, and then quantifying the remaining hLL1 antibody on the cell surface by flow cytometry. The data revealed that CD74 was internalized rapidly at 37 °C (Figure 1G), with approximately 80% of surface-bound hLL1 being internalized after 3.5 h of incubation. Collectively, these results established that CD74 represents an effective ADC target to specifically deliver drugs to CD74^+^ immune cell populations.

### 2.2. Development of a Cross-Binding and Rapidly Internalized Anti-CD74 Antibody with Rabbit Single B-Cell Screening Technology

Although CD74 is a suitable target for the development of GC-ADC, there are currently no publicly available sequences of anti-mouse CD74 antibodies that can be used for preclinical studies in murine SLE models. Therefore, we aim to develop an antibody with high cross-species binding activity and high internalization activity for conjugation with GC drugs. Based on this premise, we utilized the rabbit monoclonal antibody screening and production platform established in our laboratory. The rabbits were immunized with different CD74 antigens including recombinant human CD74-mFc protein and mouse CD74-his protein. After four rounds of immunization, high serum antibody titers were induced (Figure S1A,B), and then the CD74 antigen-specific memory B cells were isolated from rabbit PBMCs (Figure S1C). The variable regions of candidate antibodies were obtained via single cell RT-PCR, followed by high-throughput cloning and recombinant expression of candidate antibodies in CHO cells (Figure S1D). ELISA screening of CHO cell supernatants identified several high-affinity antibodies with cross-species binding activity (Figure S1E). Among these, the antibody clone 2R27, which revealed relatively higher binding activities to human, mouse, and monkey CD74, was selected for further study.

**Figure 2:**
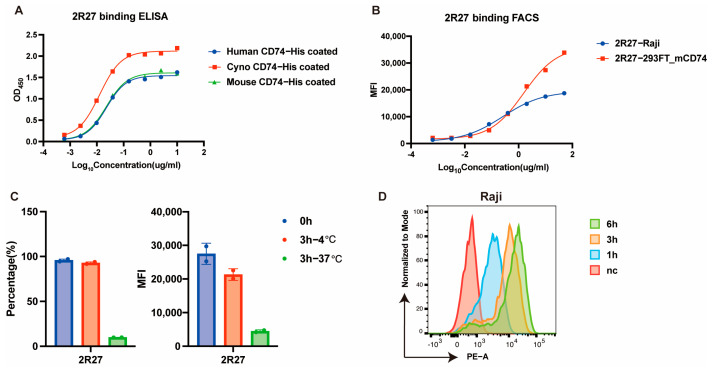
Cross-binding and internalization properties of 2R27 mAb. (**A**,**B**) Binding property of 2R27 measured by ELISA (**A**) and flow cytometry (**B**). 293FT-mCD74, HEK293FT cells with stable over-expression of mouse CD74; (**C**,**D**) Internalization assay of 2R27 measured by indirect method (**C**) and pH-sensitive probe (**D**). The results in (**C**) are presented as the means ± SEM.

ELISA and flow cytometry assays confirmed the cross-species binding activity of 2R27 (Figure 2A,B). Furthermore, we confirmed the strong internalization activity of 2R27 by measuring cell surface antibody levels at 4 °C and 37 °C (Figure 2C). We also evaluated the extent of antibody internalization into lysosomes using a pH-sensitive probe. The fluorescence signal increased during incubation (Figure 2D), indicating gradual internalization of the antibody into lysosomal compartments. These results above demonstrate that the 2R27 monoclonal antibody exhibits high cross-species binding activity, high internalization activity, and high affinity for CD74, making it suitable for conjugation with a GC drug payload to develop GC-ADC.

### 2.3. Conjugation and Evaluation of Bud-ADC

**Figure 3:**
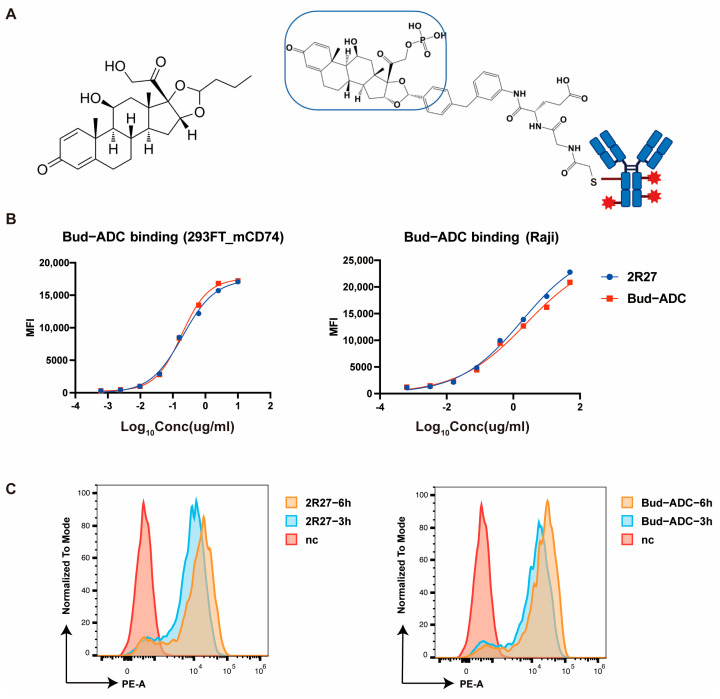
Conjugation and characterization of Bud-ADC. (**A**) Left, molecular formula of budesonide; Right, structure of Bud-ADC, the blue circle indicates the payload. (**B**) Binding property of Bud-ADC measured by flow cytometry. (**C**) Internalization assay of Bud-ADC measured by pH-sensitive probe.

Based on the 2R27 monoclonal antibody, we further developed a GC-ADC by conjugating it with a GC drug. Budesonide (Bud, Figure 3A, left) is a potent GC drug widely used in clinical settings for the treatment of asthma, allergic rhinitis, inflammatory skin diseases, colitis, and autoimmune disease [45,46,47,48,49], and its potency is approximately 1000-fold higher than that of endogenous hydrocortisone [50]. Bud is typically administered locally to target affected areas. However, Bud exhibits a short half-life, significant hepatic first-pass metabolism, and poor systemic bioavailability, and may lead to systemic side effects commonly associated with glucocorticoids under the high dosage and long-term use of Bud in patients [51]. In order to enhance the efficiency and reduce the side effects of Bud, it was selected as a payload to construct the GC-ADC. Bud was modified with a protease-sensitive linker to form a linker–payload complex, which was then conjugated to the 2R27 antibody via Cys-based coupling, as with the Abbv-154 ADC-targeting TNF developed by AbbVie [10], resulting in Bud-ADC (Figure 3A, right).

To evaluate the conjugation efficiency and determine the drug-to-antibody ratio (DAR), we performed LC-MS analysis. Compared to the 2R27 monoclonal antibody, the conjugate showed no detectable unconjugated antibody residuals (Figure S2A), and the DAR of Bud-ADC was calculated to be approximately 6.55. Additionally, size-exclusion chromatography (SEC) analysis confirmed the absence of significant protein aggregates or degradation products (Figure S2B), indicating that the antibody structure remained largely intact following budesonide conjugation.

To assess whether the binding and internalization activities of Bud-ADC were comparable to those of the monoclonal antibody, we conducted parallel comparisons with 2R27. As shown (Figure 3B,C), Bud-ADC retained binding and internalization activities similar to those of the 2R27 monoclonal antibody, demonstrating its suitability for subsequent biological functional assays.

### 2.4. Bud-ADC Suppresses Immune Cell Activation in a CD74-Dependent Manner and Exerts a Bystander Effect

**Figure 4:**
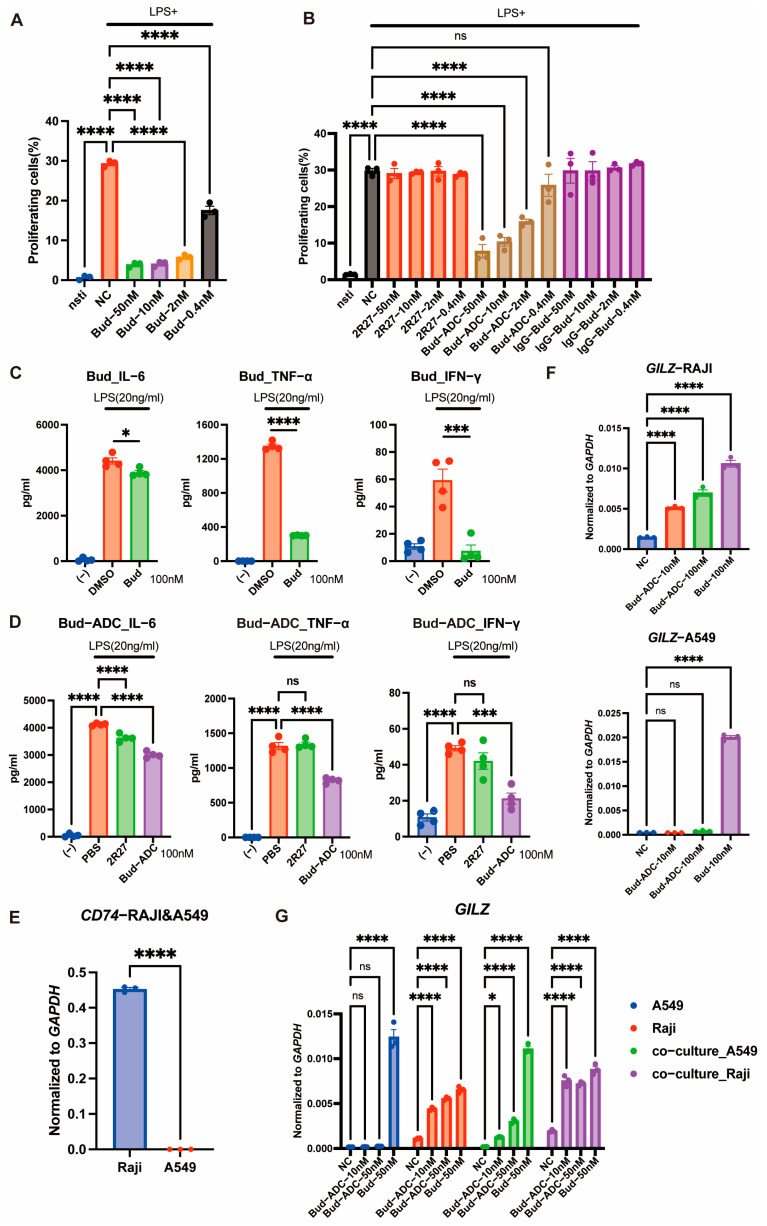
CD74-dependent immune suppression function of Bud-ADC. (**A**,**B**) Bud- (**A**) and Bud-ADC- (**B**) suppressed LPS-stimulated mouse B cell proliferation, as measured by CellTrace Violet label; (**C**,**D**) Bud- (**C**) and Bud-ADC- (**D**) suppressed LPS-stimulated cytokine secretion by human PBMCs. (**E**) The *CD74* gene RNA expression of Raji and A549 cells measured by quantitative RT-PCR. (**F**) The *GILZ* gene RNA expression of Raji and A549 cells after Bud or Bud-ADC treatment measured by quantitative RT-PCR. (**G**) *GILZ* mRNA expression levels in Raji and A549 cells cultured alone or co-cultured in a transwell system after treatment with Bud or Bud-ADC. Expression was determined by quantitative RT-PCR. The results in (**A**–**G**) are presented as the means ± SEM. Statistical analyses in (**C**,**E**) were performed with unpaired *t* test, and in (**A**,**B**,**D**,**F**,**G**) were performed with one-way ANOVA or two-way ANOVA. * *p* < 0.05, *** *p* < 0.001, **** *p* < 0.0001, “ns” indicates no significant difference.

To evaluate the biological activity of Bud-ADC, we first conducted in vitro assays. Given the broad immunosuppressive effects of glucocorticoids, we assessed the immunosuppressive function of Bud-ADC in comparison with the monoclonal antibody (mAb) and free Bud. Mouse B cells were isolated and stimulated with LPS. The results showed that Bud effectively suppressed LPS-induced B cell proliferation (Figure 4A). Bud-ADC similarly exhibited dose-dependent inhibitory effects in vitro (Figure 4B), whereas the 2R27 mAb alone and the control ADC IgG-Bud, which was produced by conjugating Bud to an isotype control antibody, showed no such activity. To further characterize its immunosuppressive activity, we evaluated Bud-ADC’s ability to inhibit pro-inflammatory cytokine secretion in LPS-stimulated human PBMCs. Both Bud and Bud-ADC significantly suppressed the expression of IL-6, TNF-α, and IFN-γ (Figure 4C,D). Although PBMCs comprise heterogeneous cell populations including T cells and NK cells with low CD74 expression, the observed efficacy of Bud-ADC suggests overlapping between CD74-high cell types and LPS-responsive populations. In contrast, the 2R27 mAb failed to inhibit cytokine secretion.

To validate target-dependent activity of Bud-ADC, we analyzed classical glucocorticoid-responsive gene *GILZ* expression in CD74-high Raji cells versus CD74-low A549 cells (Figure 4E). Bud-ADC significantly upregulated *GILZ* expression in Raji cells but showed minimal effect in A549 cells (Figure 4F), confirming CD74-dependent intracellular delivery. Free Bud induced *GILZ* in both cell lines, independent of CD74 expression. In addition, we further examined whether Bud-ADC exerts a bystander effect, which is a well-recognized property of ADCs, especially those conjugated with lipophilic payloads. Our results showed that Bud-ADC markedly increased *GILZ* expression in A549 cells co-cultured with Raji cells (Figure 4G), suggesting that Bud-ADC is able to mediate a bystander effect.

Collectively, Bud-ADC selectively delivers budesonide to CD74-expressing cells, exerting glucocorticoid-mediated transcriptional regulation and robust immunosuppressive effects, including inhibition of immune cell proliferation and pro-inflammatory cytokine secretion. Moreover, Bud-ADC may also suppress neighboring immune cells with low CD74 expression through its bystander effect.

### 2.5. Bud-ADC Effectively Alleviates Lupus Phenotypes in a Spontaneous MRL/Lpr SLE Model

After confirming the CD74-dependent immunosuppressive activity of Bud-ADC in vitro, we aimed to evaluate its therapeutic efficacy in experimental murine SLE models in vivo. We first used the spontaneous SLE model with MRL/Lpr mice. The pathogenesis of this model arises from an *Lpr* gene mutation that impairs Fas receptor expression, leading to defective lymphocyte apoptosis and severe autoimmune reaction.

**Figure 5:**
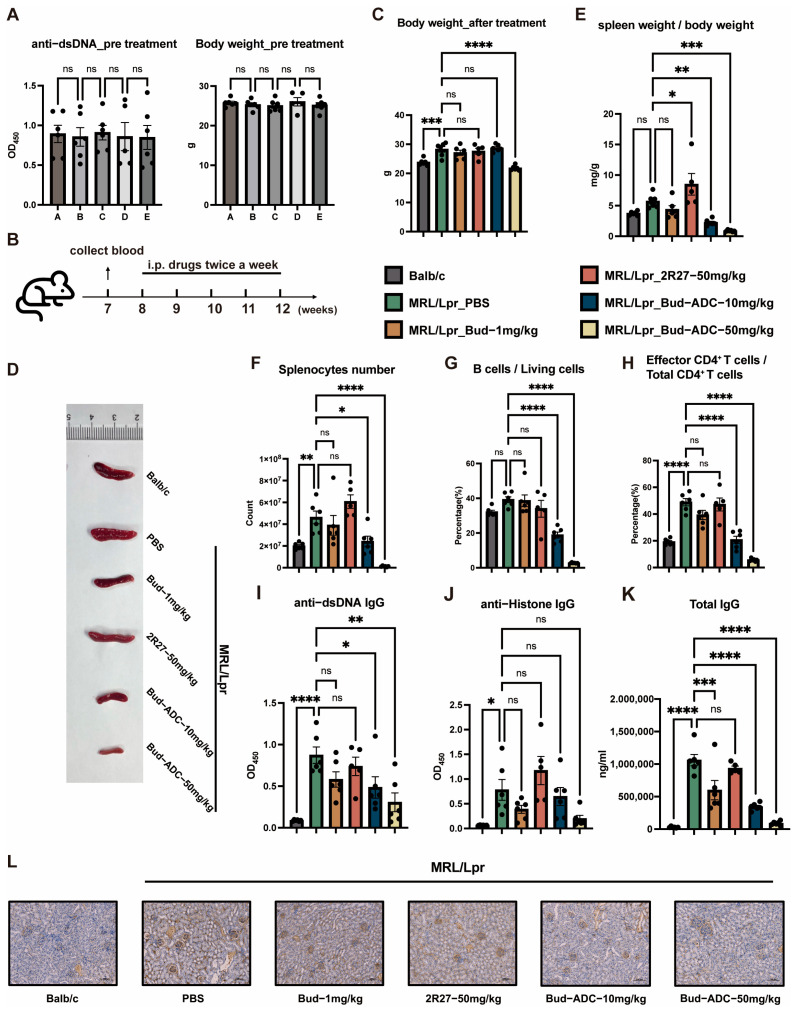
In vivo treatment of Bud-ADC in the MRL/Lpr model. (**A**) To group before treatment, the serum anti-dsDNA IgG level of mice (**left**) was measured by ELISA and body weight (**right**) was recorded. (**B**) The diagram of the experiment period of treatment. (**C**) Body weight after treatment. (**D**) Representative image of spleens of different treatment groups. (**E**) The spleen weight to body weight ratio of different treatment groups. (**F**–**H**) The splenocyte number (**F**), the proportion of B cells to the total living cells (**G**), and the proportion of effector CD4^+^ T cells to the total CD4^+^ T cells (**H**) after treatment were measured by flow cytometry. (**I**–**K**) Serum anti-dsDNA IgG (**I**), anti-Histone IgG (**J**) and total IgG (**K**) after treatment were measured by ELISA. (**L**) Representative Immunohistochemistry (IHC) images of kidney sections showing IgG deposition (bars, 100 μm). The results in (**A**,**C**,**E**–**K**) are presented as the means ± SEM (*n* = 5 or 6 mice per group). Statistical analyses were performed with one-way ANOVA. * *p* < 0.05, ** *p* < 0.01, *** *p* < 0.001, **** *p* < 0.0001, “ns” indicates no significant difference.

Serum anti-dsDNA antibody level and body weight were measured and grouping was performed based on these (Figure 5A). Treatment commenced one week later (Figure 5B). Two Bud-ADC dose groups were established: 50 mg/kg and 10 mg/kg. The equivalent dose of conjugated Bud in 50 mg/kg Bud-ADC was calculated to be about 1 mg/kg, serving as a control, alongside a 2R27 mAb treatment group (50 mg/kg). The results showed that there was no significant difference in body weight after treatment between different groups, except that the 50 mg/kg Bud-ADC group showed minor body weight reduction (Figure 5C). Splenomegaly, a hallmark of SLE, was markedly attenuated in both Bud-ADC dose groups (Figure 5D). Comparison of spleen-to-body weight ratios revealed potent immune suppression activity of Bud-ADC (Figure 5E), as even a 10 mg/kg dose reduced the ratios below those of healthy Balb/c controls, in contrast to the ineffectiveness of 1 mg/kg Bud. Flow cytometric analysis demonstrated that Bud-ADC significantly reduced spleen immune cell number (Figure 5F), consistent with spleen-to-body weight ratios. CD74-high B cell populations were selectively depleted by Bud-ADC (Figure 5G). Unexpectedly, Bud-ADC also reduced effector T cell proportions in CD4^+^ T cells (Figure 5H), suggesting indirect immunoregulatory mechanisms despite low CD74 expression in T cells.

Dose-dependent reductions in serum anti-dsDNA and anti-Histone autoantibody titers were observed (Figure 5I,J), with total IgG levels also significantly reduced (Figure 5K). Renal IgG deposition, a key driver of lupus nephritis, was alleviated by Bud-ADC (Figure 5L), demonstrating systemic therapeutic benefits. Collectively, Bud-ADC demonstrates robust efficacy in mitigating SLE-associated pathologies in MRL/Lpr mice, including autoantibody production, splenomegaly, and renal damage. Moreover, the multi targeting of CD74-high B cells, DCs and downstream T cell activation indicates its multimodal action.

### 2.6. Bud-ADC Effectively Alleviates Lupus Phenotypes in an Induced BM12 SLE Model

To further elucidate the therapeutic efficacy of Bud-ADC, we investigated its effects in another SLE model, a BM12 model, which was generated by transferring CD4^+^ T cells from BM12 mice into C57BL/6 recipients. Chronic graft-versus-host disease (cGVHD) arose from the alloreactive activation of donor CD4^+^ T cells by recipient antigen-presenting cells (APCs), resulting in SLE-like manifestations [52].

**Figure 6:**
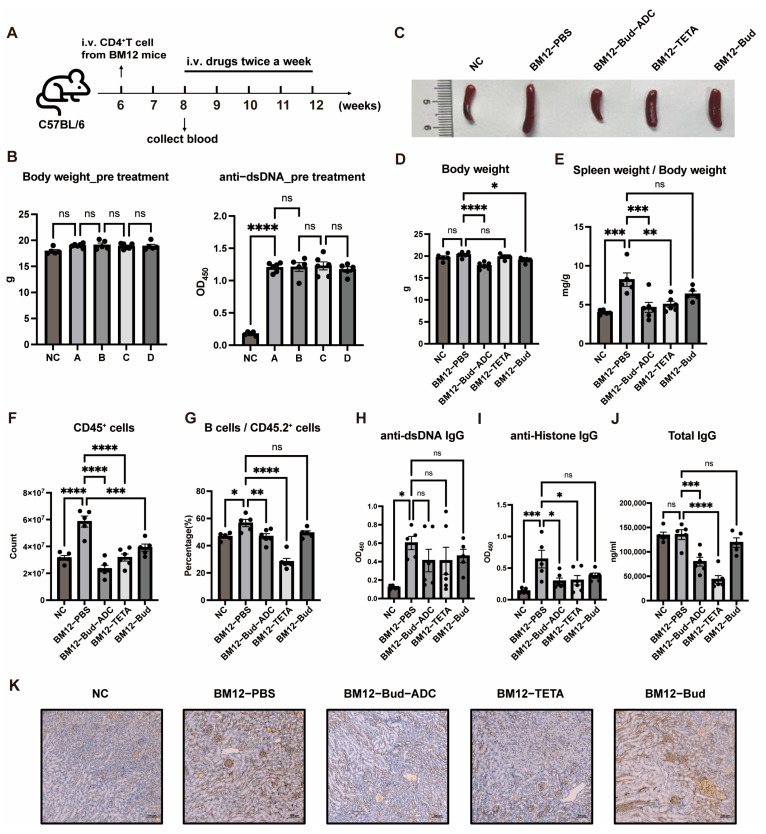
In vivo treatment of Bud-ADC in the BM12 model. (**A**) The diagram of the experiment period of model induction and treatment. (**B**) To group before treatment, the serum anti-dsDNA IgG level of mice (**right**) was measured by ELISA and body weight (**left**) was recorded. (**C**) Representative image of spleens of different treatment groups. (**D**) Body weight after treatment. (**E**) The spleen weight to body weight ratio of different treatment groups. (**F**,**G**) The count of spleen CD45^+^ cells number (**F**) and the proportion of B cells to the CD45.2^+^ cells (**G**) after treatment were measured by flow cytometry. (**H**–**J**) Serum anti-dsDNA IgG (**H**), anti-Histone IgG (**I**), and total IgG (**J**) after treatment were measured by ELISA. (**K**) Representative IHC images of kidney sections showing IgG deposition (bars, 100 μm). The results in (**B**,**D**–**J**) are presented as the means ± SEM (*n* = 5 or 6 mice per group). Statistical analyses were performed with one-way ANOVA. * *p* < 0.05, ** *p* < 0.01, *** *p* < 0.001, **** *p* < 0.0001, “ns” indicates no significant difference.

Two weeks after transfer (Figure 6A), serum anti-dsDNA antibody levels in BM12 model mice were significantly elevated (Figure 6B), confirming successful model induction with high reproducibility. Mice were grouped based on serum anti-dsDNA antibody levels and body weight (Figure 6B), followed by a 4-week treatment. Based on the therapeutic outcomes in the MRL/lpr model, where 10 mg/kg Bud-ADC demonstrated significant efficacy without notable body weight reduction, we selected a single 10 mg/kg Bud-ADC dose for this BM12 model experiment. The 2R27 mAb group was excluded due to lack of efficacy, while the Bud group (0.2 mg/kg, equivalent to the conjugated Bud of 10 mg/kg Bud-ADC) and Telitacicept (TETA, a recently approved SLE drug [41]) were included as controls.

Bud-ADC induced a slight but significant reduction in body weight (Figure 6D), whereas other groups showed no significant changes. Bud-ADC significantly decreased the spleen-to-body weight ratio (Figure 6C,E), comparable to TETA, while the Bud alone exhibited no therapeutic effect. Flow cytometric analysis revealed that all treatments reduced splenic CD45^+^ immune cell counts, with Bud-ADC showing the most pronounced reduction (Figure 6F). Both Bud-ADC and TETA significantly suppressed B cell proportions (Figure 6G), though TETA exhibited stronger inhibition, likely due to its mechanism of blocking BLyS and APRIL signaling to prevent aberrant B cell function. Notably, Bud-ADC maintained B cell proportions within normal ranges.

Serum anti-Histone antibody levels were markedly reduced by Bud-ADC and TETA (Figure 6H), while anti-dsDNA antibody levels showed a decreasing trend without statistical significance (Figure 6I). Total serum IgG levels were significantly suppressed by both treatments (Figure 6J), while Bud alone showed no efficacy. Renal IgG deposition was alleviated by both Bud-ADC and TETA (Figure 6K).

In conclusion, Bud-ADC demonstrates robust therapeutic efficacy in the induced SLE BM12 model, effectively mitigating splenomegaly, autoantibody production, and renal pathology, suggesting broad applicability across SLE models with distinct pathogenic mechanisms. Its performance parallels that of the clinically approved drug Telitacicept. These findings underscore Bud-ADC’s potential as a novel therapeutic agent for SLE, warranting further clinical investigation.

### 2.7. Bud-ADC Does Not Cause Severe Toxicity in Mice Following High-Dose Administration

**Figure 7:**
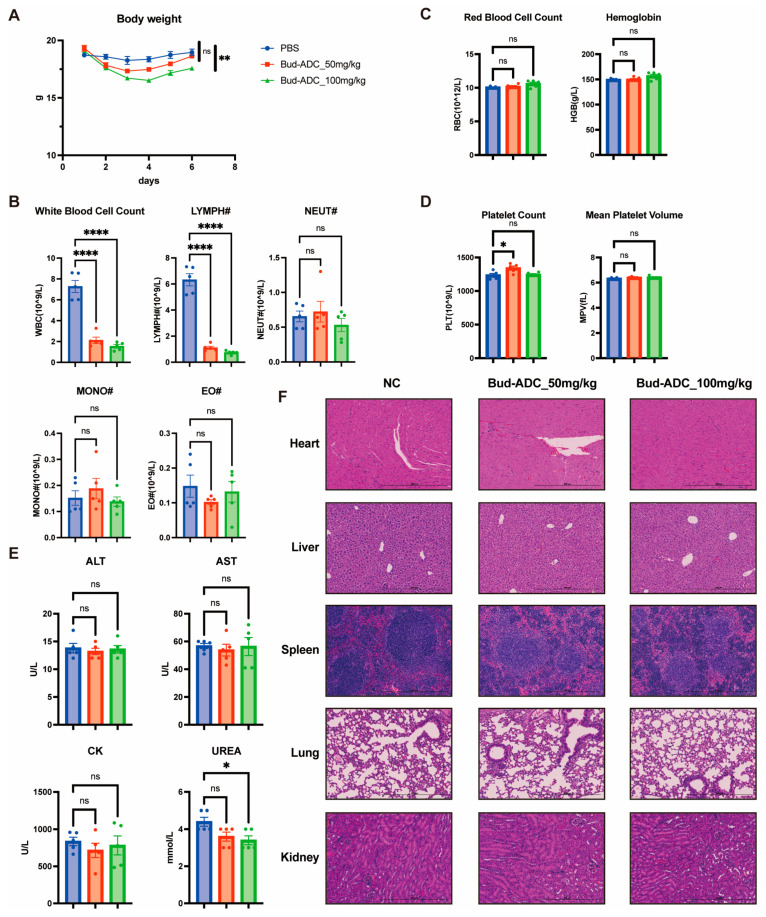
Toxicological analysis of Bud-ADC. (**A**) Changes in body weight following Bud-ADC treatment. (**B**) White blood cell counts, including subpopulations, measured 7 days after Bud-ADC administration. LYMPH#, lymphocyte count; NEUT#, neutrophil count; MONO#, monocyte count; EO#, eosinophil count. (**C**) Red blood cell counts and hemoglobin concentrations 7 days post-treatment. (**D**) Platelet counts and mean platelet volume (MPV) changes 7 days post-treatment. (**E**) Comparison of serum Alanine Aminotransferase (ALT), Aspartate Aminotransferase (AST), Creatine Kinase (CK), and UREA levels 7 days after Bud-ADC treatment. (**F**) Representative hematoxylin and eosin (H&E) staining images of major organs from each group (bars, 500 μm). The results in (**A**–**E**) are presented as the means ± SEM (*n* = 5 mice per group). Statistical analyses were performed with two-way ANOVA (**A**) and one-way ANOVA (**B**–**E**). * *p* < 0.05, ** *p* < 0.01, **** *p* < 0.0001, “ns” indicates no significant difference.

To evaluate the potential safety profile of Bud-ADC, we conducted an independent study using a single high-dose administration model. Following a single administration of Bud-ADC at 50 mg/kg or 100 mg/kg, a decrease in body weight was observed in mice. However, mice in the 50 mg/kg group gradually recovered to normal levels within one week, while those in the 100 mg/kg group also showed a trend toward recovery (Figure 7A). Hematological and serum biochemical analyses were performed seven days after the single administration. Peripheral white blood cell counts, particularly lymphocyte counts, were markedly reduced in both Bud-ADC treatment groups, which was expected since B cells physiologically express high levels of CD74 (Figure 7B). In contrast, red blood cell count, hemoglobin concentration, platelet count, and mean platelet volume were not affected by Bud-ADC treatment (Figure 7C,D). Serum biochemical indicators reflecting liver, kidney, and cardiac function also showed no evidence of acute organ toxicity (Figure 7E), even at the higher Bud-ADC dose. Consistently, histopathological examination of major organs by H&E staining revealed no structural abnormalities or tissue damage (Figure 7F).

In summary, these data indicate that apart from a reversible reduction in body weight and an on-target effect on lymphocytes, Bud-ADC did not induce apparent tissue or organ toxicity, suggesting a favorable in vivo safety profile.

## 3. Discussion

In this study, we combined the targeting capacity of anti-CD74 antibody and the profound anti-inflammation activity of budesonide by rational design of Bud-ADC to specifically deliver GC drugs into CD74-high immune cell populations, trying to minimize the side effects of GC to other tissues. Based on the rabbit mAb screening system using single cell RT-PCR, we successfully obtained the expected antibody clone 2R27, which shows high affinity with human, mouse, and monkey CD74 and can be internalized rapidly, making it possible to simultaneously evaluate the antibody in a murine model but also put it forward for clinical use. The Bud-ADC was produced by conjugating budesonide to the 2R27 antibody with a cleavable linker. We demonstrate that the Bud-ADC exhibits good physical and chemical properties, and the affinity and internalization abilities were not affected by the conjugation. The Bud-ADC exerted its biological function in a CD74-dependent manner, and exhibited strong immune suppressive function in vitro. Moreover, murine lupus models of different pathologies can be well resolved by Bud-ADC. The potency of Bud-ADC when treating in vivo is much stronger than the equivalent dosage of budesonide, supporting the idea that targeted delivery of budesonide to immune cells is a promising strategy, mainly due to its longer half-life and better stability in vivo as a result of its conjugation with the antibody and the targeted delivery by an anti-CD74 antibody. Additionally, in comparison with Telitacicept, a newly approved biologic for SLE that primarily suppresses B cell immune responses, Bud-ADC targets CD74 to broadly inhibit multiple immune cell types, which is theoretically expected to achieve greater efficacy and has demonstrated comparable therapeutic effects in SLE animal models. Lastly and importantly, Bud-ADC exhibits an overall favorable in vivo safety profile.

As mentioned earlier, the development of ADC is still mainly limited in the field of cancer treatment to targeting the delivery of cytotoxic drugs to tumor cells. However, the payload of ADC can be diverse, including inhibitors or agonists of some signaling pathways, PROTAC, siRNA and so on, which means that it is not necessary to induce cell death. Blocking or stimulating the key signaling pathway of target cells may be enough to treat non-cancer disease. This enables ADCs to treat diseases which are not as fatal but still lack effective and safe treatment, especially when the drugs used cause side effects because of an unexpected and non-targeting effect. There are approved drugs focusing on treating SLE by targeting B cells, inhibiting type I IFNs mainly produced by pDCs or other strategies. Although they are reported to be effective, they still only affect single cell types or pathways, which may not be strong enough in some circumstances in clinical use. Conversely, Bud-ADC may exert more effective treatment by the broad inhibition of multiple immune cell types.

A previous study has explored the feasibility of conjugating GC drugs to an anti-CD74 antibody [8]. They found the ADC to be less effective at first, so they inhibited the membrane-penetrating ability of the GC drug payload to make it function in the cell rather than diffuse to the extracellular space. However, we think it is better to retain the membrane-penetrating ability of GC drug payload to enable the bystander effect of ADC, which may explain the inhibitor effect of the Bud-ADC to T cells observed in the SLE murine model treatment assay. The Bud-ADC may cause the enrichment of budesonide in the immune organs such as the spleen and lymph nodes by targeting release in CD74-high immune cells like B cells, and diffusing to neighboring CD74-low immune cells like T cells, achieving even broader suppression.

Although the Bud-ADC exhibits promising potential to treat SLE, and/or other autoimmune diseases, there remains work to be done before clinical use. Firstly, it is not clear whether Bud-ADC can avoid the side effects caused by long-term traditional GC drug treatment, which is one of the expected benefits of our primary design. The evaluation of these side effects in a murine model may be challenging, but we may be able to detect downstream gene expression regulated by glucocorticoid receptors in different tissues. Secondly, underlying toxicity has not been excluded. In fact, the spleen became abnormally small after 50 mg/kg Bud-ADC treatment in the murine model, not only proving its powerful efficacy, but also hinting at the risk of over-suppression of the immune system at high dosage, so it is necessary to determine a safe and effective dosage before proposing this treatment.

In summary, to overcome the limitation of GC drugs in clinical use, we have developed the Bud-ADC to deliver budesonide targeted to CD74-high immune cells, avoiding effects on normal tissues. The Bud-ADC retains the binding affinity and internalization capacity of the anti-CD74 antibody 2R27, and exhibits potent immunosuppressive activity both in vitro and in vivo. Notably, treatment with Bud-ADC effectively alleviates disease symptoms in multiple murine models of SLE. Additional work needs to be carried out, as mentioned above, to study the clinical availability of Bud-ADC. Nevertheless, Bud-ADC represents a feasible strategy for the design of ADCs aimed at treating autoimmune and other non-cancer diseases, particularly when the primary pathogenic cell type can be specifically targeted by an antibody.

## 4. Materials and Methods

### 4.1. Immunization of Rabbit and Screening of Anti-CD74 Monoclonal Antibody by Single Cell RT-PCR

To generate the cross-reactive anti-CD74 antibody, rabbits were immunized with recombinant human CD74-mouse Fc tag protein and mouse CD74-His tag protein (recombinant expressed and purified by Biointron (Shanghai, China) or purchased from Novoprotein (Suzhou, China)) at week 0, week 4, week 8, and week 12. During the immunization period, rabbit serum was collected regularly to determine the anti-CD74 antibody titer. After immunization, rabbit PBMCs were isolated by density gradient centrifugation. To acquire the variable region sequence of anti-CD74 antibody, single CD74-antigen specific memory B cells (gated as FITC (IgM/CD4/CD8/Pan-T)-/APC-IgG+/biotinylated human CD74 and mouse CD74 protein with streptavidin-BV421+) were sorted into a 96-well plate containing 10 μL RNase-free water (Sangon Biotech, Shanghai, China) and 20 U RNase inhibitor (Promega, Madison, WI, USA) in each well by a Sony MA900 flow separator (Sony, Japan). The variable region sequence was acquired by single cell RT-PCR followed by nested PCR to amplify. The PCR products were sequenced and then analyzed using the National Center for Biotechnology Information (NCBI)-IgBLAST (https://www.ncbi.nlm.nih.gov/igblast/, accessed on 10 October 2022) and IMGT/V-QUEST (http://www.imgt.org/IMGT_vquest, accessed on 10 October 2022). The selected PCR products were cloned into the corresponding vector and the antibodies were expressed by CHO cells. The rabbits were housed at the Shanghai Tengda Rabbit Industry Professional Cooperative. The immunization was performed in accordance with the relevant regulations of the Shanghai Tengda Rabbit Industry Professional Cooperative.

### 4.2. Expression and Purification of Antibodies

The DNA fragments encoding VH and VL CDS regions of the antibodies were cloned into vectors Abvec-HIgG1-VH for the heavy chain, and Abvec-HIgG1-VK or Abvec-HIgG1-Vλ for the light chain. The heavy chain and light chain plasmids were cotransfected into Expi CHO cells according to the manufacturer’s instructions. Then, the transfected cells were cultured and the antibody-containing supernatant was collected. The antibodies were purified using Protein A Sepharose (Cytiva, NY, USA), followed by size-exclusion chromatography (AKTA PURE 25M, GE, Uppsala, Sweden).

### 4.3. Cell Culture

HEK293FT, Raji, Jurkat, and A549 cells were obtained from the American Type Culture collection. HEK293FT and A549 cells were cultured in DMEM medium (Yuanpei, Shanghai, China) containing 10% fetal bovine serum (FBS, Vazyme, Nanjing, China) and 1% penicillin/streptomycin (P/S, Yuanpei, Shanghai, China). Raji and Jurkat cells were cultured in RPMI1640 medium (Yuanpei, Shanghai, China) containing 10% FBS and 1% P/S. HEK293FT cells stably expressing different isoforms of mouse or human CD74 were generated by lentivirus infection and were cultured as HEK293FT cells. CHO cells (Thermo Fisher, Waltham, MA, USA) were cultured in Expi CHO expression medium (Thermo Fisher, Waltham, MA, USA). Human PBMCs were purchased from hys-bio company (Shanghai, China) and were cultured in RPMI1640 medium containing 10% FBS, 1% P/S, 1% glutamine (Gbico, Grand Island, NY, USA), and 1% HEPES (Gbico, Grand Island, NY, USA). Mouse B cells were isolated from the spleens of 6–8 weeks old female C57BL/6 mice by mouse B cell isolation kit (Stemcell, Vancouver, BC, Canada), according to the manufacturer’s instructions.

### 4.4. Conjugation and Analysis of Bud-ADC

Bud-conjugated ADC was prepared as previously described by Shangyaojiaolian [44]. Briefly, the antibody was partially reduced by incubating with 10× molar concentrations of tris-(2-carboxyethyl)-phosphine (TCEP, MCE, NJ, USA) at 37 °C for 1 h. Then, an eight-fold molar excess of linker–payload was added to the reduced antibodies for 1 h at room temperature. The ADCs were subsequently purified by dialysis to remove unconjugated linker–payload.

The drug-to-antibody ratio (DAR) was determined by liquid chromatography-mass spectrometry (LC-MS, Waters, H-class bio + Xevo G2-S QTof, MA, USA). Briefly, the antibody–drug conjugate (ADC) sample (2 mg/mL, 100 μL) was deglycosylated with PNGase F (2 μL) at 37 °C for 4 h. Following incubation, the sample was centrifuged at 12,000× *g* for 5 min. Subsequently, 90 μL of the supernatant was transferred to an LC-MS vial for analysis.

The purity of the Bud-ADC was assessed by size-exclusion chromatography coupled with high-performance liquid chromatography (HPLC-SEC, Agilent, 1260 Infinity II, CA, USA). A sample (100 μL, 0.5 mg/mL) was loaded into an HPLC vial and placed in the autosampler. Chromatographic separation was performed using a BioCore SEC-300 column (Nanochrom, Suzhou, China) with isocratic elution consisting of 0.2 M potassium dihydrogen phosphate and 0.25 M potassium chloride (pH adjusted to 6.95 ± 0.05), mixed with isopropanol (85:15, *v*/*v*).

### 4.5. Enzyme-Linked Immunosorbent Assay (ELISA)

To determine the binding properties of the purified anti-CD74 antibodies, Bud-ADC, the antibody containing supernatant of CHO cells, and the serum of immunized rabbit, mouse CD74-His protein, or human CD74-mouse Fc protein (diluted to 5 ug/mL by PBS, Yuanpei, Shanghai, China) was coated to the surface of a 96-well plate (Thermo Fisher, Waltham, MA, USA) kept overnight at 4 °C; then, the coated plate was blocked by 2% bovine serum albumin (BSA, Sigma-Aldrich, St. Louis, MO, USA) in PBS-Tween 20 (PBST) for 1 h at room temperature. Antibodies or serum were serially diluted and incubated in the wells for 2 h at room temperature. Then, the plate was washed three times by PBST, and the anti-human or anti-rabbit Fc antibody conjugated with HRP (Sigma-Aldrich, St. Louis, MO, USA) was used to detect antibodies bound to the coated protein, followed by incubation with TMB substrate (Beyotime, Haimen, China), which was stopped by adding 1 mol/L HCl. The absorbance at 450 nm was recorded by a plate reader (Bio-Tek Epoch 2, Winooski, VT, USA).

Human PBMCs were plated in a 96-well plate and pre-treated by budesonide (MCE, NJ, USA or Bud-ADC followed by stimulating with LPS (MCE, NJ, USA) for 24 h. The supernatant were collected and the concentration of cytokine IL-6, TNFα, and IFNγ was measured using an ELISA kit (Thermo Fisher Invitrogen, Waltham, MA, USA) according to the manufacturer’s instructions.

For analysis of mouse serum autoantibody and total IgG, mouse serum was prepared by orbital blood sampling, and diluted by PBST with 2% BSA. The anti-dsDNA and anti-Histone IgG were measured as described [53]. Generally, dsDNA (MCE, NJ, USA) and Histone (Sangon Biotech, Shanghai, China) were diluted by PBS and coated to the surface of a 96-well plate overnight at 4 °C. After blocking, diluted serum was added to the wells and incubated for 2 h at room temperature. Then, the plates were washed three times with PBST followed by detection using anti-mouse IgG-HRP (R&D, Minneapolis, MN, USA). The signal was developed by adding TMB substrate (Beyotime, Haimen, China) and stopped by 1 mol/L HCl. The serum total IgG was measured by a Mouse IgG (Total) Uncoated ELISA Kit (Invitrogen, Waltham, MA, USA) according to the manufacturer’s instructions.

### 4.6. Internalization Assay

The internalization properties of anti-CD74 antibodies were determined by FACS. Briefly, high-CD74 cells were incubated with anti-CD74 antibodies at 4 °C, followed by washing three times to remove unbound antibodies. Then, the cells were incubated at 4 °C or 37 °C for different times. The antibodies remaining on the cell surface were detected by anti-human IgG-AF488 (Thermo Fisher Invitrogen, Waltham, MA, USA).

Alternatively, the anti-CD74 antibodies were incubated with pH-sensitive probes Incucyte ^®^ Human Fabfluor-pH Orange Antibody Labeling Reagent (Sartorius, Göttingen, Germany), according to the manufacturer’s instructions. Then, the complex was added to high-CD74 cells, followed by further incubation at 37 °C for different times. The fluorescence intensity in the PE panel indicated the internalization properties of anti-CD74 antibodies.

### 4.7. Flow Cytometric Analysis FACS

The binding properties of anti-CD74 antibodies and Bud-ADC were also measured by flow cytometry. Generally, high-CD74 cells were pre-stained with FVS780 (BD Biosciences, Franklin Lakes, NJ, USA) and plated in a V-bottom 96-well plate followed by incubation with diluted antibodies or ADCs. After being washed, the cells were stained by anti-human IgG-AF488 (Thermo Fisher Invitrogen, Waltham, MA, USA). The stained cells were analyzed using a Sony ID7000 flow cytometer (Sony, Japan).

The proliferation of mouse B cells was analyzed using CellTrace Violet (Thermo Fisher Invitrogen, Waltham, MA, USA), according to the manufacturer’s instructions.

### 4.8. Bystander Effect Assay

To evaluate the bystander effect of Bud-ADC, a transwell co-culture system (Corning, NY, USA) was employed. Briefly, Raji cells were seeded in the upper chamber of a transwell insert with a 0.4 μm pore membrane, which allows for the diffusion of small molecules but prevents cell migration. A549 cells were seeded in the lower wells of a 12-well plate. Bud-ADC or free budesonide at the indicated concentrations were added to the upper chamber. After 48 h of treatment, Raji and A549 cells from the corresponding groups were collected separately for RNA extraction.

### 4.9. RNA Isolation and Quantification

Total RNA of different cell types was extracted using TRIzol (Thermo Fisher Invitrogen, Waltham, MA, USA) and reverse transcribed into cDNA with HiScript III RT SuperMix for qPCR (Vazyme, Nanjing, China). Then, qPCR was performed by using a Taq Pro Universal SYBR qPCR Master Mix (Vazyme, Nanjing, China) and detected using an ABI QuantStudio™ 6 PCR detection system (Thermo Fisher, Waltham, MA, USA). The sequences of primers used in this article are listed below.

Human *GAPDH*: forward, 5′-AGATCCCTCCAAAATCAAGTGG-3′, reverse, 5′-GGCAGAGATGATGACCCTTTT-3′;

Human *CD74*: forward, 5′-CCATCCTGGTGACTCTGCTC-3′, reverse, 5′-CAGGCTTGGGAGGCTTGG-3′;

Human *GILZ*: forward, 5′-TCTAATGCTACTGCGCCCTG-3′, reverse, 5′-CCCTGCCTTCACGAAACAGA-3′.

### 4.10. MRL/Lpr Model

All the mice were maintained under specific pathogen-free conditions at the Animal Care Facility of the CEMCS (Shanghai, China). The animal care and use procedures complied with the guidelines of the CEMCS.

The 6-week-old female MRL/Lpr mice were purchased from Shanghai SLAC Laboratory Animal Limited Liability Company (Shanghai, China), and the Balb/c mice were used as a control. The mice were grouped by body weight and serum anti-dsDNA antibody level followed by intraperitoneally treating with different drugs two times a week for 4 weeks. The mice were sacrificed 24 h after the last drug treatment. Body and spleen weight were measured. Serum, spleen, and kidney were collected. Kidney was fixed in 4% paraformaldehyde (PFA).

The spleen single cell suspension was prepared for staining with the following antibodies, CD45.2-PE (BD Biosciences, NJ, USA, 560695), CD4-PE-CY7 (Invitrogen, 25-0041-82), CD44-FITC (Invitrogen, 11-0441-85), CD62L-BV605 (BD Biosciences, 563252), B220-eF506 (Invitrogen, 69-0452-82), CD138-BV421 (Biolegend, San Diego, CA, USA, 142507), IgD-AF700 (Biolegend, 405730), and CD27-APC (Biolegend, 124211), after being pre-stained with FVS780 (BD Biosciences, 565388). The stained splenocytes were analyzed by a Beckman CytoFlex3 flow cytometer (Suzhou, China) and all flow cytometry data were analyzed with FlowJo V10 (BD Biosciences, NJ, USA).

### 4.11. BM12 Model

The BM12 transgenic mice were kindly provided by Prof. Wei Lv (Shanghai Institute of Nutrition and Health, Chinese Academy of Sciences). The BM12 SLE-like model was induced as previously reported [52]. Briefly, the CD4+ T cells were isolated from the spleen and lymph nodes of 6–8-week-old BM12 mice by negative purification (Stemcell mouse CD4+ T cell isolation kit) and counted. Then, the cells were intravenously injected into 6–8-week-old C57BL/6 mice, 8 × 10^6^ cells per mouse. Two weeks after transfer, the serum anti-dsDNA IgG was measured to make sure that the model was successfully induced, and the mice were grouped according to body weight and anti-dsDNA IgG level. Then, the mice were treated with different drugs intravenously two times a week for 4 weeks. The mice were sacrificed 24 h after the last drug treatment and analyzed as in the MRL/Lpr model, except for a difference in the FACS panel.

The splenocytes were stained with the following antibodies, CD45.2-PE (BD Biosciences, 560695), CD4-PE-CY7 (Invitrogen, 25-0041-82), CD44-FITC (Invitrogen, 11-0441-85), CD62L-BV605 (BD biosciences, 563252), B220-eF506 (Invitrogen, 69-0452-82), CD138-BV421 (Biolegend, 142507), IgD-AF700 (Biolegend, 405730), and CD27-APC (Biolegend, 124211), after being pre-stained with FVS780 (BD biosciences, 565388).

### 4.12. Toxicological Analysis of Bud-ADC

Eight-week-old female C57BL/6 mice were purchased from Shanghai SLAC Laboratory Animal Co., Ltd. (Shanghai, China), and grouped according to body weight. On day 1, the mice were administered 50 mg/kg or 100 mg/kg Bud-ADC, or PBS as a control, via intravenous injection. Body weights were recorded daily until day 6. On day 7, mice were sacrificed, and blood samples were collected via retro-orbital bleeding. Complete blood counts were performed using an XN-1000V (B1) hematology analyzer (SYSMEX, Kobe, Japan), and serum biochemical parameters were analyzed using a VITROS 4600 automatic biochemical analyzer (Ortho, VT, USA). Major organs (heart, liver, spleen, lung, and kidney) were collected and fixed in 4% paraformaldehyde (PFA) for 24 h. Hematoxylin and eosin (H&E) staining was performed on paraffin-embedded sections following standard procedures by Servicebio Co., Ltd. (Wuhan, China).

### 4.13. Immunohistochemistry (IHC)

For histopathological analysis of inflammation, kidney was fixed in 4% (PFA) for 24 h and then dehydrated and embedded in paraffin. Immunohistochemistry (IHC) was conducted on paraffin sectioned samples following standard IHC procedure with antigen recovery by Servicebio Co., Ltd. (Wuhan, China). The tissue sections were stained with anti-mouse IgG-HRP (R&D, MN, USA). Images were acquired using Aixo Scan Z1 (Zeiss, Jena, Germany).

### 4.14. Statistical Analysis

Statistical data were analyzed with GraphPad Prism 9 (GraphPad Software, CA, USA). The results are presented as the means  ±  SEMs, and *p*-values  <  0.05 were considered to indicate statistical significance. Differences in the data were analyzed with Student’s *t* test, one-way ANOVA, or two-way ANOVA, as described in the text. Data are representative of two or three independent experiments.

## Data Availability

The datasets used and/or analyzed during the current study are available from the corresponding author on reasonable request.

## Supplementary Materials

The following supporting information can be downloaded at https://www.mdpi.com/article/10.3390/ijms262311761/s1.

## Abbreviations

The following abbreviations are used in this manuscript:

GCs	Glucocorticoid drugs
SLE	Systemic lupus erythematosus
Bud	Budesonide
DCs	Dendritic cells
pDCs	Plasmacytoid DCs
MHC-II	Major Histocompatibility Complex Class II
IFN	Interferon
RT-PCR	Reverse Transcription Polymerase Chain Reaction
PBMCs	Peripheral Blood Mononuclear Cells
ELISA	Enzyme-Linked Immunosorbent Assay
CHO	Chinese Hamster Ovary cells
DAR	Drug-to-antibody ratio
LC-MS	Liquid chromatography–mass spectrometry
SEC	Size-exclusion chromatography
LPS	Lipopolysaccharide
mAb	monoclonal antibody
IL-6	Interleukin-6
TNF-α	Tumor Necrosis Factor-α
cGVHD	Chronic graft-versus-host disease
APCs	Antigen-presenting cells
TETA	Telitacicept
