# Supplementary material for: CD74-Targeting Antibody–Drug Conjugate Enhances Immunosuppression of Glucocorticoid in Systemic Lupus Erythematosus
Source: Int J Mol Sci. 2025 Dec 4;26(23):11761. doi: 10.3390/ijms262311761 (PMC12692655; doi:10.3390/ijms262311761)
Supplement: Supplementary file 1 [file ijms-26-11761-s001.zip › ijms-3852649-supplementary.pdf]

Supplementary Material

CD74-Targeting Antibody-Drug Conjugate Enhances Immunosuppression of Glucocorticoid in Systemic Lupus Erythematosus

Qizhen Du[1,#], Shengtao Yao[2,#], Yuying Huang[1], Jia Zhang[1], Wangmo Sonam[3], Xiao Lu[1], Jichao Yang[3], Shipeng Cheng[1], Ran Wang[4], Jiefang Xu[4], Liyan Ma[1], Yu Liu[2], Guanghao Wu[2], Jing Zhang[2], Xuelei Wang[5], Wei Lv[5], Zhiyang Ling[1], Chunyan Yi[1,*] and Bing Sun [1, 3,*]

[1] Key Laboratory of Multicellular Systems, CAS Center for Excellence in Molecular Cell Science, Shanghai Institute of Biochemistry and Cell Biology, University of Chinese Academy of Sciences, Chinese Academy of Sciences, 320 Yueyang Road, Shanghai, 200031 China

[2] Shanghai Pharmaceuticals Holding Co.,ltd. Halei Road 898, Shanghai, 201203 China

[3] School of Life Science and Technology, ShanghaiTech University, Shanghai, 201210 China

[4] Division of Life Sciences and Medicine, University of Science and Technology of China, Hefei, China

[5] Shanghai Institute of Nutrition and Health, Chinese Academy of Sciences; University of Chinese Academy of Sciences, 320 Yueyang Road, Shanghai, 200031 China

[#] These authors contributed equally: Qizhen Du, Shengtao Yao.

* Correspondence: bsun@sibs.ac.cn, Tel.86-21-64034171; cyyi@sibs.ac.cn.

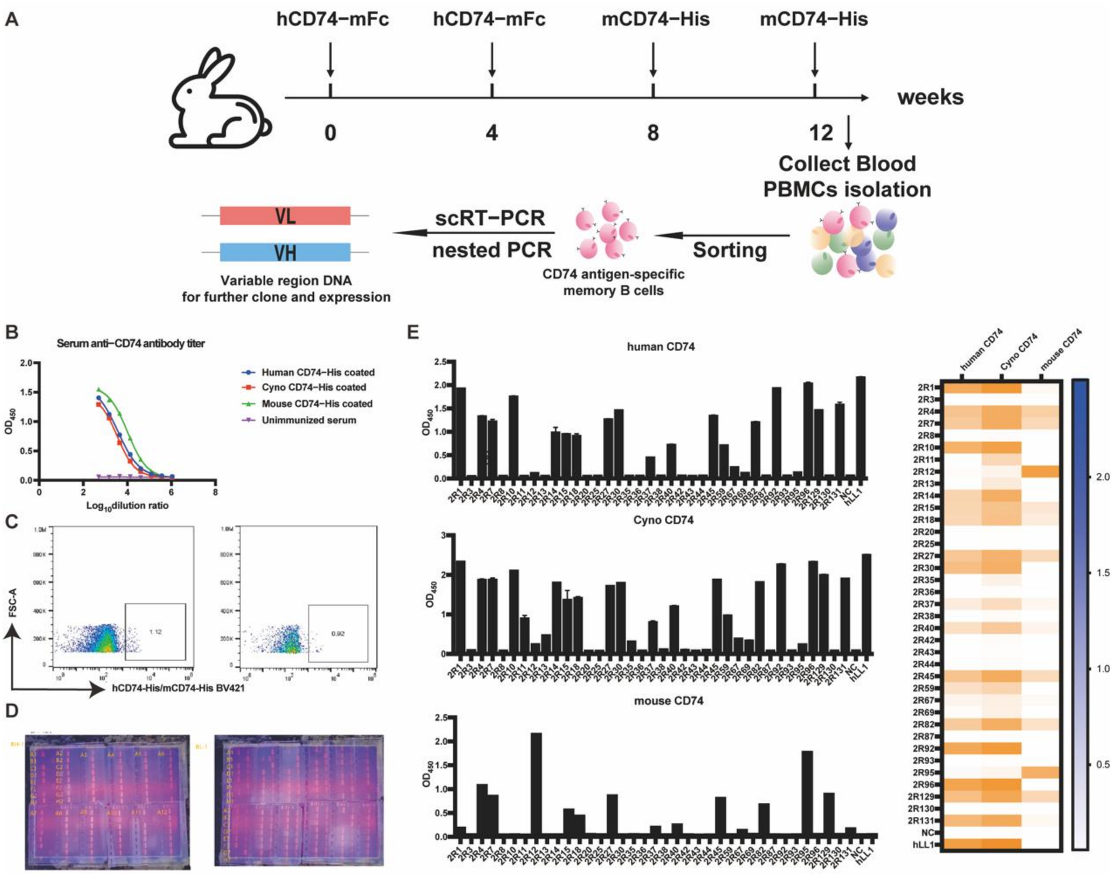

Figure S1. Immunization of rabbits and screening of cross-binding monoclonal antibodies by single cell RT-PCR. (A) The diagram of the immunization period; (B) The serum anti-CD74 antibody titer detected by ELISA; (C) CD74 antigen-specific memory B cell sorting by flow cytometry; (D) Representative image of high through clone of variable region of anti-CD74 antibodies; (E) The binding property of anti-CD74 antibody candidates measured by ELISA.

A

2R27						
Name	D0	D2	D4	D6	D8	DAR
Mass(Da)	142884	/	/	/	/	/
Intensity	10454	/	/	/	/	0.00
Theoretical molecular weight: 143157.2782						

2R-27-Bud (Bud-ADC)												
Name	D0	D1	D2	D3	D4	D5	D6	D7	D8	D9	D10	DAR
Mass(Da)	142884	143759.9	144635.7	145511.6	146387.4	147263.3	148139.1	149015	149890.8	150766.7	151642.5	/
Intensity	0	0	2030	0	1702	0	4189	1350	7118	1995.00	0.00	6.55

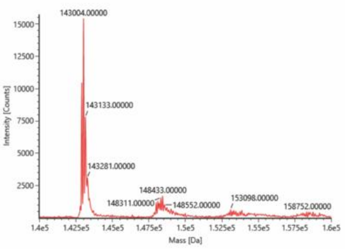

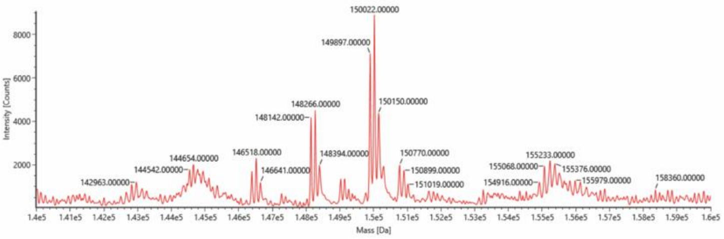

B

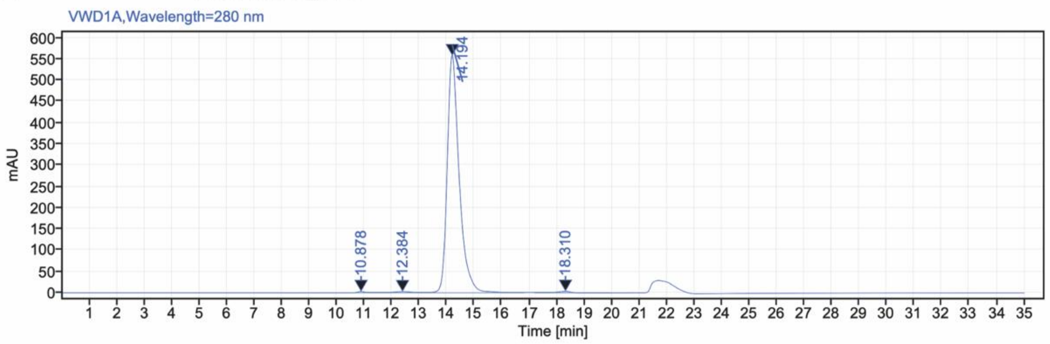

#	RT	Width(50%)	Area	Height	Area%	Resolution	Tail Factor	Symmetry	N
1	10.9	0.3	92.0	3.0	0.5		1.9	0.3	6609
2	12.4	0.9	145.7	2.7	0.8	1.5	1.0	1.0	1054
3	14.2	0.4	16995.4	558.4	97.7	1.6	1.4	0.7	5577
4	18.3	0.5	161.6	3.5	0.9	5.0	0.7	2.5	6880

Figure S2. Conjugation and characterization of Bud-ADC. (A) LC-MS analysis of 2R27 mAb (left) and Bud-ADC (right); (B) HPLC-SEC analysis of Bud-ADC.
